# Editorial: Dietary patterns in cancer prevention and survival

**DOI:** 10.3389/fnut.2024.1478256

**Published:** 2024-09-02

**Authors:** Raul Zamora-Ros, Esther Molina-Montes

**Affiliations:** ^1^Unit of Nutrition and Cancer, Cancer Epidemiology Research Program, Catalan Institute of Oncology (ICO), Bellvitge Biomedical Research Institute (IDIBELL), Barcelona, Spain; ^2^Department of Nutrition and Food Science, University of Granada, Granada, Spain; ^3^Institute of Nutrition and Food Technology “José Mataix, ” Biomedical Research Center, University of Granada, Parque Tecnológico de la Salud, Armilla, Granada, Spain; ^4^Instituto de Investigación Biosanitaria ibs.GRANADA, Granada, Spain; ^5^CIBERESP (Epidemiology and Public Health), Institute of Health Carlos III (ISCIII), Madrid, Spain

**Keywords:** dietary pattern, cancer, cancer prevention, cancer survival, epidemiological studies

Cancer is a major societal, public health, and economic problem worldwide. It is a leading cause of death worldwide, accounting for nearly 10 million deaths in 2020 (1). While advancements in medical research, including early diagnosis and better personalized treatments, have led to improved survival rates for all cancer types, its global burden is still rapidly growing (1). Although some individuals are at higher risk due to non-modifiable risk factors, between 30%−40% of all cancer cases are estimated to be preventable through healthy lifestyles, including healthy diets. However, little is known on the impact of these preventive measures on cancer survival. In 2018, a report from the World Cancer Research Fund and the American Institute for Cancer Research (2) promoted ten cancer prevention recommendations on diet and nutrition, which are also extendable to improving cancer survival. But characterizing a healthy diet is not easy, since foods and nutrients are not consumed alone and, therefore, they can interact with each other.

Over the past decade, dietary pattern analysis has emerged as an alternative and complementary approach to evaluating the relationship between diet and cancer prevention and survival (3, 4). Instead of looking at individual nutrients or foods, dietary pattern analysis examines the relationships with the overall diet. Conceptually, dietary patterns represent a broader picture of food and nutrient consumption, may provide stronger risk estimates with disease risk, and can be more easily translated into dietary guidelines.

In this Research Topic, we are providing 16 peer-reviewed manuscripts on the associations between dietary patterns (both a priori and a posteriori) and cancer risk and survival. Six of them were meta-analyses investigating the associations with Mediterranean diet (Zhu Q. et al.), nutritional status evaluated by the CONUT score (Liu et al.), and food groups overall (Qi et al.), and in particular, fruits and vegetables (Yao et al.), red and processed meats (Sun et al.), and ultra- processed foods (Lian et al.). According to these studies, high adherence to the Mediterranean diet is associated with a 29% reduction in gastric cancer risk, high (Zhu Q. et al.), a high intake of dietary fiber reduces overall cancer mortality (Yao et al.), and the intake of fruits, vegetables, alcohol, tea, and coffee is associated with a lower risk of both renal cell carcinoma and bladder cancer (Qi et al.). However, processed and red meat intake was linked to a higher renal cell carcinoma risk (Qi et al.), whereas the consumption of these foods was not related to pancreatic cancer risk in the meta-analysis by Sun et al.. Besides, the consumption of ultra-processed foods was found to increase the risk of colorectal, colon, and breast cancer (Lian et al.). With regard to gastric cancer patient's nutritional status, the meta-analysis of Liu et al. showed that a poor nutritional status or low CONUT score leads to a worse stomach cancer prognosis. In addition, another study evaluating the impact of the nutritional status on the patient's outcome proposed two other tools [Patient-Generated Subjective Global Assessment (PG-SGA) and Nutrition Risk Screening 2002 (NRS-2002)] for malnutrition screening (Chen X. et al.).

Furthermore, three of the studies evaluated several dietary factors using Mendelian randomization analysis, an approach that uses genetic variants associated with a dietary factor exposure to estimate the causal relationship between these variables and cancer risk and prognosis. Results of these studies showed that higher genetic predispositions to intake of dried fruit and oily fish are linked to a reduced risk of breast cancer and its subtypes (Wang et al.), that of cheese, dried fruit, and beer appeared to be associated with lung cancer risk or its subtypes (Yan et al.), whereas there was no significant association between coffee or caffeine consumption and the risk or prognosis of endometrial cancer (Chen Z. et al.).

Five of the included studies investigated the association between a priori dietary patterns (e.g., oxidative stress exposure, dietary total antioxidant capacity, diabetes risk reduction diet, microbial diet, and dietary approaches to stop hypertension eating pattern–DASH) and the risk of several types of cancers in large prospective or retrospective studies. Specifically, two studies highlighted the cancer-preventive effects of antioxidant-related dietary patterns: a higher Oxidative Balance Score (OBS) integrating nutrient antioxidants was associated with a lower risk of colorectal cancer in women but not in men in a large prospective study involving over 1,000 cancer patients (Gu et al.), and an antioxidant-rich diet was significantly linked to a reduced risk of head and neck cancer in an Iranian case-control study (Toorang et al.). Dietary patterns related to the prevention of cardiovascular disease, the DASH diet, and diabetes, were inversely associated with lung cancer risk (Zhu Z. et al.), and with head and neck cancer (Wu et al.), respectively. Also, a higher adherence to a sulfur microbial diet, which is related to the enrichment of sulfur-metabolizing gut bacteria, was associated with an increased risk of colorectal adenoma in older adults (Xiao et al.). These three studies were prospective and evidenced differences in the associations by smoking status.

Finally, the last one studied the associations of maternal a posteriori dietary patterns and the risk of leukemia in children in a case control study from Mexico, where a vegetable-rich diet was found to reduce the risk of this disease in infants (Muñoz-Aguirre et al.).

We sincerely hope that this Research Topic of works from around the world will provide high quality epidemiological evidence and bring some light to the complex relationships between diet and cancer prevention and survival.

## References

[1] Bray F Laversanne M Sung H Ferlay J Siegel RL Soerjomataram I. Global cancer statistics 2022: GLOBOCAN estimates of incidence and mortality worldwide for 36 cancers in 185 countries. CA Cancer J Clin. (2024) 74:229–263. 10.3322/caac.21834 38572751

[2] World Cancer Research Fund/American Institute for cancer Research. Diet, Nutrition, Physical Activity and Cancer: a Global Perspective. Continuous Update Project Expert Report 2018. Available at: www.dietandcancerreport.org

[3] Steck SE Murphy EA. Dietary patterns and cancer risk. Nat Rev Cancer. (2020) 20:125–138. 10.1038/s41568-019-0227-4 31848467

[4] Castro-Espin C Agudo A. The role of diet in prognosis among cancer survivors: a systematic review and meta-analysis of dietary patterns and diet interventions. Nutrients. (2022) 14:348. 10.3390/nu14020348 35057525 PMC8779048

